# Dexmedetomidine may decrease the bupivacaine toxicity to heart

**DOI:** 10.1515/med-2021-0311

**Published:** 2021-07-15

**Authors:** Zhousheng Jin, Fangfang Xia, Tingting Lin, Yaoyao Cai, Hongfei Chen, Yuelan Wang

**Affiliations:** Department of Anesthesiology and Perioperative Medicine, Shandong Qianfoshan Hospital, Cheeloo College of Medicine, Shandong University, Jinan, Shandong Province, 250014, China; Department of Anesthesiology, The First Affiliated Hospital, Wenzhou Medical University, Wenzhou, Zhejiang Province, 325000, China

**Keywords:** dexmedetomidine, cardiac tolerance, bupivacaine, PI3K/Akt pathway, ZO-1

## Abstract

**Objective:**

The purpose of our study was to explore the effect of dexmedetomidine on cardiac tolerance to bupivacaine.

**Method:**

Human coronary endothelial cells were used to establish *in vitro* model. They were randomly divided into control (Con) group, dexmedetomidine (Dex) group, bupivacaine (Bupi) group, dexmedetomidine + bupivacaine group (DB group), and dexmedetomidine + bupivacaine + PI3K inhibitor (DB-inhibitor) group. Cell activity was measured by Cell counting kit-8 (CCK-8). Transwell was used to detect cell permeability. Western blotting was used to detect the protein expression of related factors.

**Results:**

There were no notable differences in cell activity among the five groups (*P* > 0.05). Dexmedetomidine significantly reduced the permeability of endothelial cells to bupivacaine and increased the protein expression of Zonulaoeeludens-1 (ZO-1) (*P* < 0.01). However, the aforementioned effects of dexmedetomidine were disappeared after the addition of PI3K inhibitors. Furthermore, Dex and DB markedly increased the protein expression of PI3K, p-Akt, and p-PTEN in comparison with Con group (*P* < 0.001), but there was no significant difference in p-PTEN among DB-inhibitor, Con, and Bupi groups (*P* > 0.05).

**Conclusion:**

Dex reduced Bupi-induced vasopermeability through protein expression of ZO-1 and PI3K/Akt pathway, which may lead to the decrease of Bupi-induced cardiotoxicity.

## 1 Introduction

With the development of ultrasound technology, the application of regional nerve blocks in the clinic has been increased [1]. However, regional nerve block requires the use of large doses of local anesthetics, and there is a risk of local anesthetic toxicity [2]. Local anesthetic toxicity can cause systemic toxicity, even cardiac toxicity such as arrhythmia or cardiac arrest in severe cases [3,4]. Therefore, how to improve the tolerance of heart to local anesthetic toxicity is the focus of clinical anesthesiologists. Bupivacaine is one of the local anesthetics with long-acting amide, which has strong anesthetic effect and good postoperative analgesic effect, and it is widely used in clinical anesthesia. However, its cardiac toxicity is the strongest in the same class of amide local anesthetics [5]. So bupivacaine, as a representative of amide local anesthetics, is often used in the study of cardiac toxicity of local anesthetics.

Dexmedetomidine, a selective α2 adrenergic agonist, plays an antisympathetic and antianxiety, sedative, and analgesic action through inhibiting the discharge of neurons by acting on the brain and spinal cord α2-adrenoreceptor (α2-AR) [6]. Thus, dexmedetomidine is widely used in regional block anesthesia and plays an important role in the effects of local anesthetics [7,8]. Hanci et al. found that SD rats, which were pretreated with dexmedetomidine, had enhanced cardiac tolerance to bupivacaine toxicity [9]. It has also been reported that in the presence of cardiotoxic symptoms, the plasma bupivacaine concentration in the clonidine pretreatment group is more than two times higher than that in the control group, but there is no difference in cardiac concentration [10]. It is suggested that dexmedetomidine can enhance cardiac tolerance to bupivacaine toxicity, but the mechanism is unknown, which may be related to the effect of dexmedetomidine on vasopermeability and distribution of bupivacaine.

Therefore, we hypothesized that dexmedetomide could reduce vasopermeability to bupivacaine and increase cardiac tolerance to bupivacaine toxicity. We placed endothelial cells on a semi-permeable membrane to simulate the vascular wall. We established a bupivacaine toxicity model and measured the permeability of vascular endothelial cells to bupivacaine.

## 2 Materials and methods

### 2.1 Chemicals and antibodies

Dexmedetomidine hydrochloride injection was obtained from Jiangsu Xinchen Pharmaceutical Co., Ltd. (Lianyungang, China). Bupivacaine hydrochloride injection was bought from Shanghai Hefeng Pharmaceutical Co., Ltd. (Shanghai, China). Human coronary artery endothelial cells (HCAECs) and endothelial cell medium (ECM) were obtained from ATCC cell bank (ScienCell companies, USA). Cell counting kit-8 (CCK-8) cell proliferation–toxicity detection reagent was bought from DOJINDO Japanese company (Japan). Bicinchoninic acid assay (BCA) kits were obtained from ZSGB Biotech Co., Ltd. (Beijing, China). All kinds of antibodies, including anti-zonula occludens-1 (anti-ZO-1), anti-Phosphoinositide 3-kinase (anti-PI3K), anti-protein kinase B (anti-p-Akt), anti-phospho-Akt (anti-Akt), anti-phosphatase and tensin homolog deleted on chromosome ten (anti-PTEN), and anti-phospho-PTEN (anti-p-PTEN), were purchased from Cell Signaling Technology (Beverly, MA, USA). Rabbit anti-actin polyclonal antibody was bought from Abcam Technology (Cambridge, UK). Anti-glyceraldehyde-3-phosphate dehydrogenase (anti-GAPDH) antibody was purchased from ZSGB Biotech Co., Ltd. (Beijing, China). Unless specified, all other reagents are obtained from Sigma Chemical Co. (St. Louis, MO, USA).

### 2.2 Cell culture and grouping

HCAECs were cultured with ECM containing 10% fetal bovine serum (FBS; Sangon Biotech, Inc., Shanghai, China) and 2 mM l-glutamine and antibiotics (100 U/mL penicillin and 100 U/mL streptomycin) in an incubator at 37°C in 5% CO_2_. When cells reached the logarithmic phase of cell growth, they were divided into five groups: (1) control group (Con group): cells were not treated; (2) dexmedetomidine group (Dex group): cells were treated with 200 μM dexmedetomidine for 2 h; (3) bupivacaine group (Bupi group): cells were treated with 1,000 μM bupivacaine for 2 h; (4) dexmedetomidine + bupivacaine group (DB group): cells were treated with dexmedetomidine and bupivacaine for 2 h; (5) dexmedetomidine + bupivacaine + PI3K inhibitor group (DB-inhibitor group): cells were treated with dexmedetomidine (200 μM), PI3K inhibitor LY294002 (20 μM), and bupivacaine (1,000 μM) for 2 h. After culturing with different drugs, cells and culture media were collected for follow-up experiments.

### 2.3 Measurement of vascular permeability *in vitro*

Endothelial permeability assays were performed with endothelial cells grown on Transwell polycarbonate membrane inserts (6.5 mm in diameter, 3 μm in pore size; Costar Inc., Suzhou, China) [11]. Cell suspension (2 × 10^6^ cells) was added to the upper chambers of the Transwell apparatus. After cells were confluent (usually 24–48 h after seeding), the medium was carefully replaced by the serum-free medium. Different concentrations of bupivacaine or bupivacaine/dexmedetomidine or bupivacaine/dexmedetomidine/PI3K inhibitor LY294002 was added to the upper chamber. After 2 h, the inserts were carefully lifted, and the medium in the lower chamber was stirred. The concentration of bupivacaine in the lower chamber was then measured by liquid chromatography/mass spectrometry (Agilent Technologies, Inc, Santa Clara, California) [12]. In DB inhibitor group, monolayers were incubated with PI3K inhibitor LY294002 for 2 h before the addition of bupivacaine or dexmedetomidine.

### 2.4 Cell viability measurement by CCK-8 assay

After culturing with different drugs, sterilized phosphate buffer saline (PBS; Shanghai Yuanmu Biotechnology Co., Ltd., Shanghai, China) was used to fill the cells to the edge. A total of 100 μL CCK-8 solution was added to each well, and the cells were incubated at 37°C for 2 h. Absorbance was measured at 450 nm using an enzyme-labeled meter (Thermo Fisher Scientific, Waltham, MA, USA).

### 2.5 Western blotting

The total protein of cells was homogenized in cold RIPA buffer (Beyotime Institute of Biotechnology, Shanghai, China) containing protease inhibitor (100×) and phosphatase inhibitor (Sangon Biotech Inc., Shanghai, China). The protein concentration was determined using the BCA kit according to the introduction. SDS-PAGE (BIO-RAD Co., California, USA) was used to separate protein by electrophoresis. After that, the protein was transferred to the nitrocellulose membrane (Pall Co., NY, USA). We indicated the different range of membrane cutting according to PageRuler Prestained Protein Ladder (10–170 kDa; Thermo, #26616). The membrane with phosphorylated protein was blocked with bovine serum albumin (Sangon Biotech Inc., Shanghai, China), while that with nonphosphorylated protein was blocked with 5% nonfat dried milk (Sangon Biotech Inc., Shanghai, China) for 1 h. Then, the membrane was cultured with primary antibodies overnight at 4°C. The membrane was placed in secondary antibodies for 1 h at room temperature. After that, ECL luminescence solution was added, and the gray value of the bands was automatically developed and read by Molecular Imager Chemi Doc XRS (BIO-RAD Co., California, USA) and JS-780 automatic gel imaging analysis systems. GAPDH was used as the internal control.

### 2.6 Statistical analysis

Data analysis was performed using SPSS software version 19.0 (SPSS Inc., Chicago, IL). All data were expressed as mean ± standard deviation (mean ± SD). Statistical significance between groups was compared by one-way analysis of variance (ANOVA) with LSD/Bonferroni’s *post hoc* comparison test. *P* < 0.05 was considered statistically significant.


**Ethic Approval:** This study was approved by the ethics committee and institutional review board of Shandong Qianfoshan Hospital, Cheeloo College of Medicine, Shandong University.
**Informed consent:** All patients submitted written informed consent before the start of the therapy.

## 3 Results

### 3.1 Effect of dexmedetomidine or bupivacaine on cell viability of HCAECs

**Figure 1:**
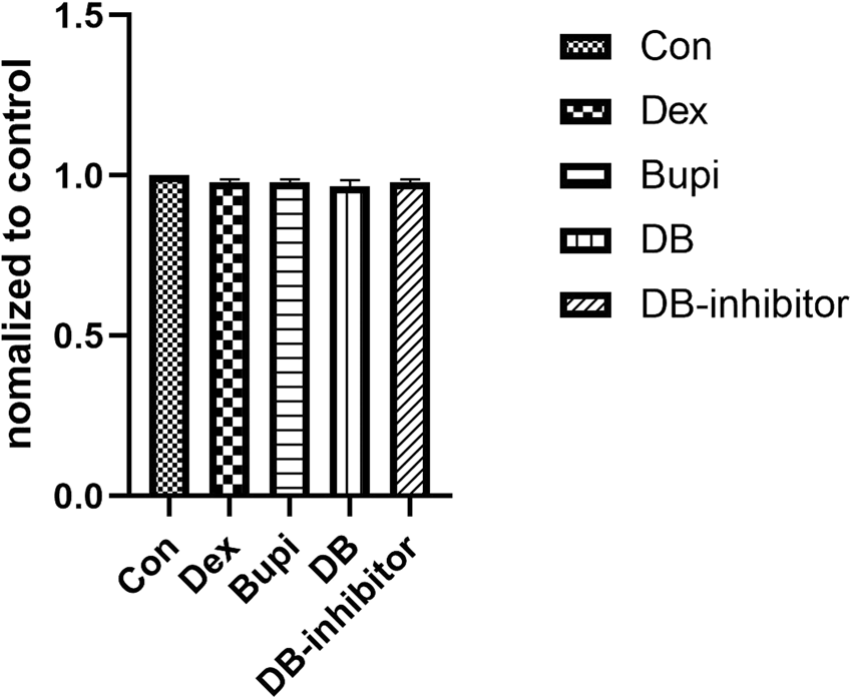
CCK-8 assay for detection of cell viability. Data were expressed as mean ± SD.

We first used the CCK-8 assay to detect the effects of dexmedetomidine and bupivacaine on cell viability of HCAECs. As shown in Figure 1, there was no significant difference in cell viability of HCAECs among the five groups (*P* = 0.738).

### 3.2 Effect of dexmedetomidine or bupivacaine on permeability of HCAECs

**Figure 2:**
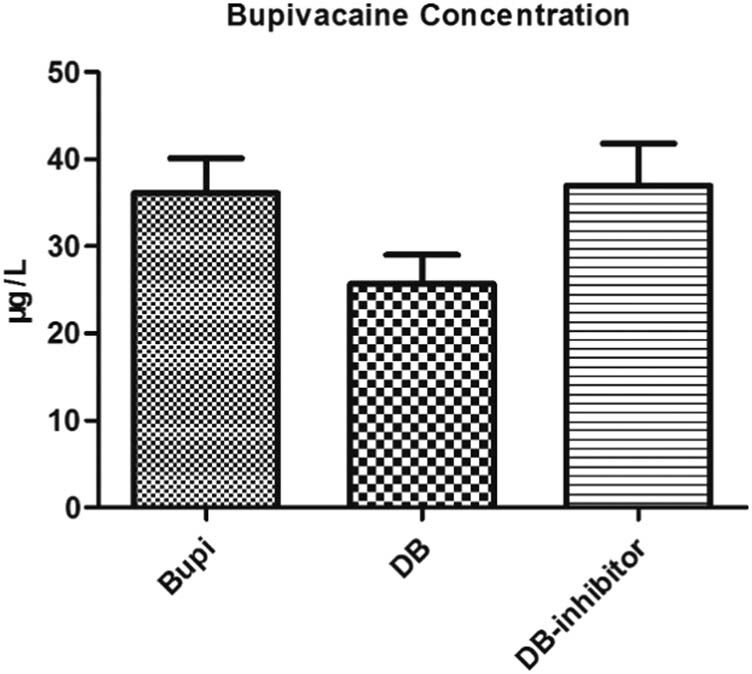
Bupivacaine concentration in Transwell lower chamber at 2 h. Bupivacaine concentration in DB group was significantly lower than that in other two groups (Bupi vs DB, *P* = 0.002, DB-inhibitor vs DB, *P* = 0.001). There was no statistical difference between Bupi group and DB inhibitor group (*P* = 0.751). *P < 0.05 vs DB group.

As shown in Figure 2, transendothelial transport of bupivacaine across the endothelial cell monolayer was decreased in DB group compared with Bupi group (*P* = 0.002). This effect of dexmedetomidine was reversed after the addition of PI3K inhibitors. The concentration of bupivacaine in DB inhibitor group was higher than DB group (*P* = 0.001). However, there was no difference in permeability between bupivacaine group and DB inhibitor group (*P* = 0.751).

### 3.3 Effect of dexmedetomidine or bupivacaine on the expression of permeability protein ZO-1

**Figure 3:**
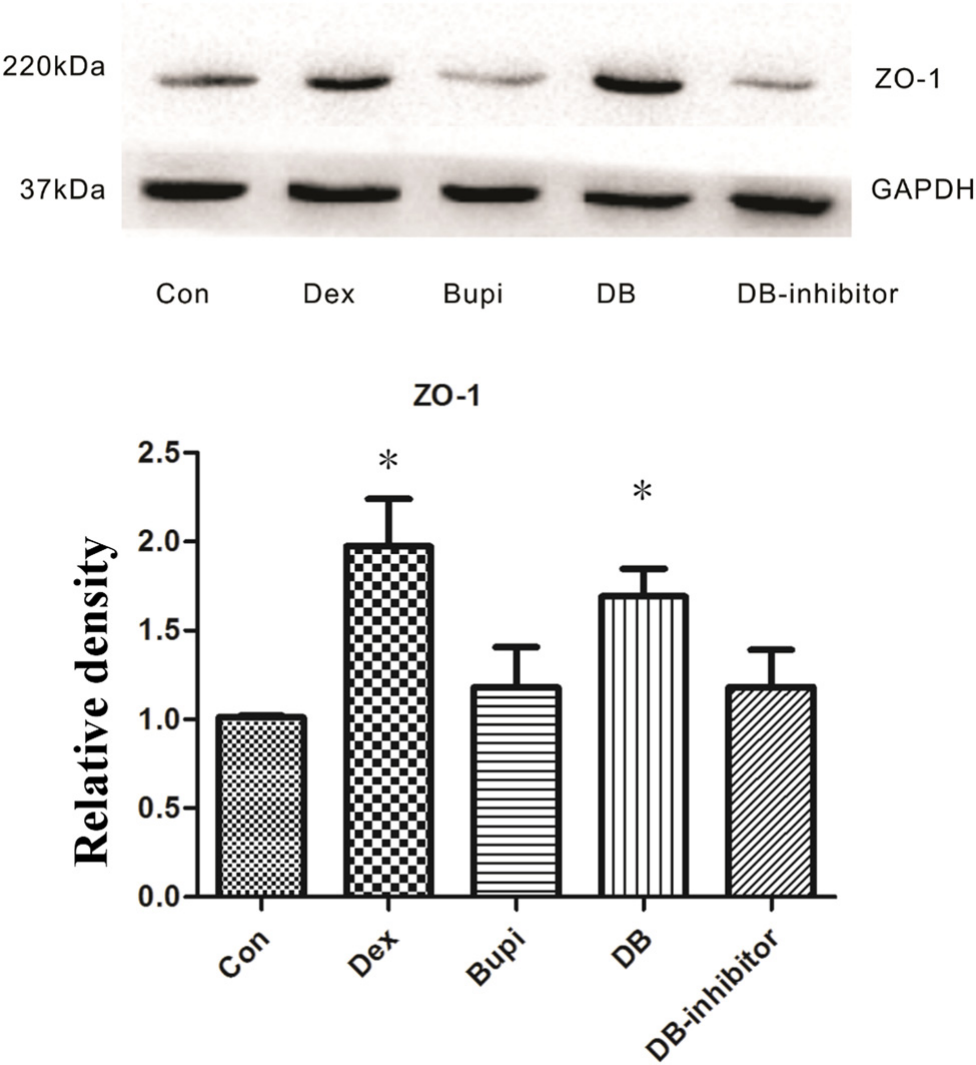
ZO-1 protein expression. The protein expression levels of ZO-1 in DB group and Dex group was higher than that in other three groups. *P < 0.05 vs Con, Bupi, and DB inhibitor.

As shown in Figure 3, there were statistically significant differences in the protein expression levels of ZO-1 among the five groups (*P* < 0.001). The protein expression of ZO-1 was significantly higher in Dex group and DB group than that in the control group (Dex vs Con, *P* < 0.001; DB vs Con, *P* < 0.001). Meanwhile, the protein expression of ZO-1 was notably higher in Dex group or DB group than in Bupi group (Dex vs Bupi, *P* < 0.001; DB vs Bupi, *P* < 0.001). However, there was no significant difference in protein expression of ZO-1 between Bupi and Con groups (Bupi vs Con, *P* = 0.052). Similarly, there was no significant difference in protein expression of ZO-1 between DB inhibitor and control groups (DB-inhibitor vs Con, *P* = 0.091).

### 3.4 Effect of dexmedetomidine or bupivacaine on the protein expression of PI3K, p-Akt and p-PTEN

**Figure 4:**
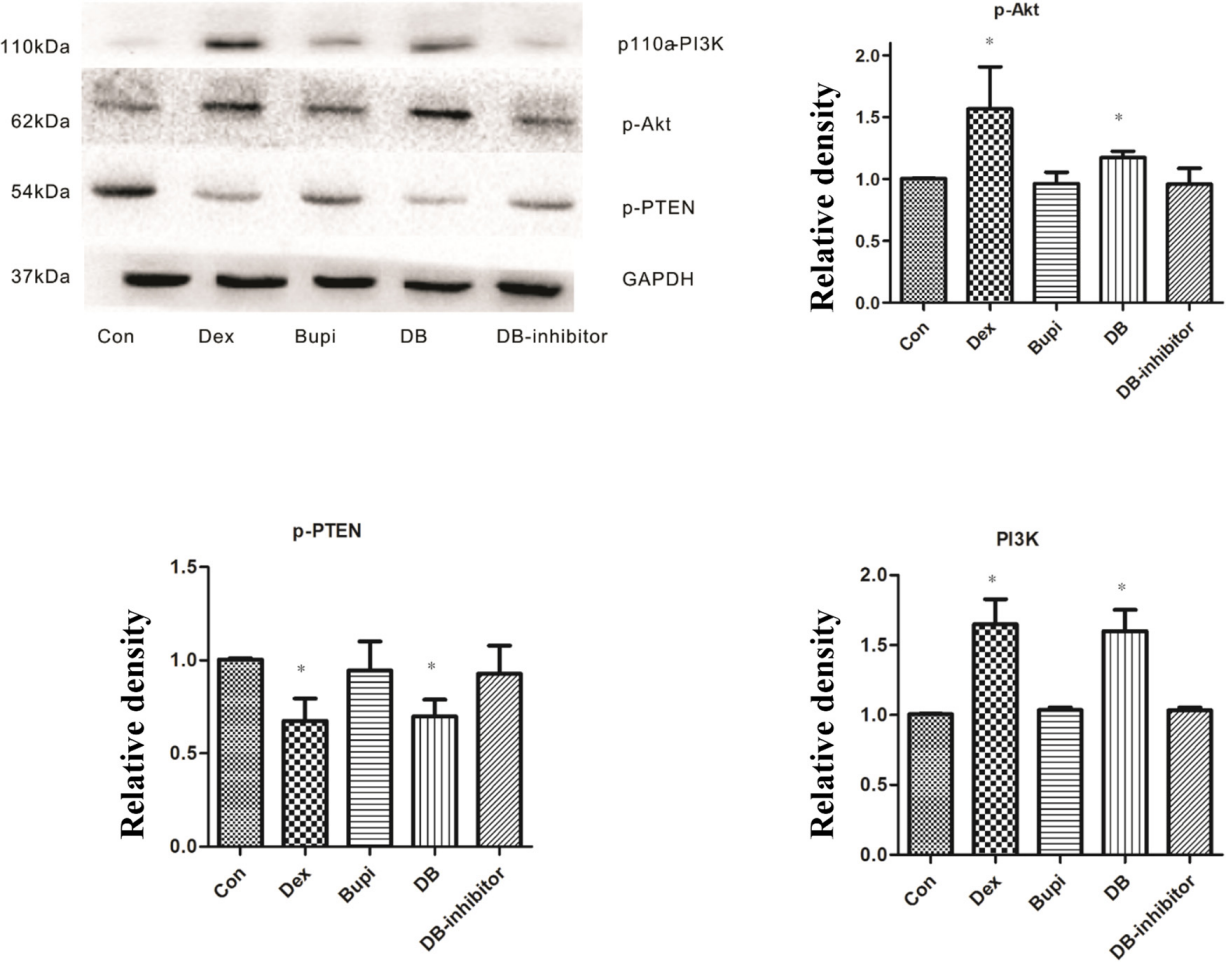
The protein expression of related factors in the PI3K/Akt pathway. PI3K and p-Akt protein levels in DB group and Dex group were higher than that in other three groups. The expression of p-PTEN in DB group and Dex group was lower than that in other three groups. There was no significant difference in the expression of Akt and PTEN between the two groups. *P < 0.05 vs Con, Bupi, and DB inhibitor.

There were statistically significant differences in the expression of PI3K, p-Atk, and p-PTEN between the five groups (*P* < 0.001). The protein expression levels of PI3K and p-Akt were markedly increased in Dex and DB groups in comparison with those in the control group (Dex vs Con, *P* < 0.05; DB vs. Con, *P* < 0.05), while the protein expression levels of PI3K and p-Akt were not increased in Bupi and DB-inhibitor groups (Bupi vs. Con, *P* > 0.05; DB-inhibitor vs. Con, *P* > 0.05; Figure 4). Furthermore, the protein expression of p-PTEN was notably decreased in Dex and DB groups when compared to the control group (Dex vs Con, *P* < 0.001; DB vs Con, *P* = 0.001), while there was no significant difference among Bupi, DB inhibitor, and Con groups (Bupi vs. Con, *P* = 0.683; DB-inhibitor vs. Con, *P* = 0.383; Figure 4).

## 4 Discussion

In this study, we found that dexmedetomidine can activate the PI3K/Akt pathway under the bupivacaine toxicity background, increase the protein expression of ZO1, and reduce the vasopermeability to bupivacaine in HCAECs.

Zonulaoeeludens-1 (ZO-1) is a protein related to tight junction (TJ), which is discovered in 1986 [13]. In recent years, ZO-1 has been found to be related to regulating and maintaining the function of epithelial fence and barrier. In most cases, as long as ZO-1 is damaged, the function of TJ will change accordingly. Therefore, ZO-1 is often used as an indicator to observe the barrier function and the permeability function of various tissues.

In our study, endothelial cells were spread on a semi-permeable membrane to simulate the vascular wall, and then bupivacaine or a mixture of bupivacaine and dexmedetomidine was added to the container to observe the penetration of bupivacaine through the simulated vascular wall. It was found that dexmedetomine reduced the amount of bupivacaine permeating through this vascular wall. These results suggested that dexmedetomidine can reduce the permeability of blood vessels, thereby reducing the distribution of bupivacaine from blood vessels to organ tissue. Meanwhile, the results showed that the protein expression of ZO-1 was significantly increased in the DB group in comparison with the bupivacaine group, suggesting that dexmedetomidine may reduce vascular permeability to bupivacaine by increasing the expression of ZO-1. The aforementioned results explain the research results of Hanci et al. from the molecular level, that is, dexmedetomidine reduces vascular permeability by increasing the expression of ZO-1 protein, “locking” bupivacaine in the blood vessels, and may ultimately increase the cardiac tolerance to bupivacaine toxicity. Tanaka et al. found that dexmedetomidine decreased the convulsive potency of both bupivacaine and levobupivacaine in rats [14]. Similar results were obtained in the renal ischemia–reperfusion model, where dexmedetomidine increased protein expression of ZO-1 and protected the distant lung [15]. The aforementioned research results also confirm the results of our study.

In the rat brain injury experiment, the protein expression of related factors in the PI3K/Akt pathway was measured [14]. The experimental results showed that the brain trauma model inhibited the protein expression of ZO-1 and related factors in the PI3K/Akt pathway. However, dexmedetomidine could reverse this effect, and the effect disappeared after the addition of the PI3K inhibitor. The results suggested that dexmedetomidine could reduce the blood–brain barrier permeability by activating the PI3K/Akt pathway to achieve brain protection. Zemljic-Harpf et al. also proved that the PI3K/Akt pathway can increase the protein expression of ZO-1 [16]. These results suggested that dexmedetomidine may regulate ZO-1 protein expression through the PI3K/Akt pathway, thereby reducing vascular permeability. In this study, the effect of dexmedetomidine on the PI3K/Akt pathway under the background of bupivacaine toxicity was observed, and the results showed that dexmedetomidine activated the PI3K/Akt pathway by increasing the protein expression levels of PI3K, p-Akt, and p-PTEN. The results were similar to those of the previous studies. Then, after the addition of PI3K inhibitor, the effect of dexmedetomidine on increasing the protein expression of ZO-1 disappeared, and the effect of dexmedetomidine on vascular permeability also disappeared. This suggested that in the case of bupivacaine-induced toxicity, dexmedetomidine can reduce vascular permeability by activating the PI3K/Akt pathway to increase the protein expression of ZO-1.

There were also some limitations of this study. We only explored the mechanism of dexmedetomidine on reducing vascular permeability of HCAECs from the cellular level. In addition, the concentrations of Dex and Bupi are higher than those under clinical conditions. Further studies using clinically relevant concentrations of Dex and Bupi are needed to investigate the effects of Dex in detail. We found that dexmedetomidine may increase cardiac tolerance to bupivacaine toxicity through regulating HCAECs ZO-1 protein expression by the PI3K/Akt pathway. In the future, we will study the effects of different concentrations of dexmedetomidine in animals and the underlying mechanisms by knocking-down/overexpressing target genes in HCAECs. We will continue to test this in animals

## 5 Conclusion

In the bupivacaine toxicity cell model, dexmedetomidine regulates ZO-1 protein expression through the PI3K/Akt pathway to reduce cardiac vascular permeability and may ultimately enhance the cardiac tolerance to bupivacaine toxicity.
